# Exploring the association between EEG microstates during resting-state and error-related activity in young children

**DOI:** 10.21203/rs.3.rs-2865543/v1

**Published:** 2023-05-02

**Authors:** Armen Bagdasarov, Kenneth Roberts, Denis Brunet, Christoph M. Michel, Michael S. Gaffrey

**Affiliations:** Duke University; Duke University; University of Geneva; University of Geneva; Duke University

**Keywords:** Error-related negativity, Performance monitoring, EEG microstates, Resting-state networks, Source localization, Children

## Abstract

The error-related negativity (ERN) is a negative deflection in the electroencephalography (EEG) waveform at frontal-central scalp sites that occurs after error commission. The relationship between the ERN and broader patterns of brain activity measured across the entire scalp that support error processing during early childhood is unclear. We examined the relationship between the ERN and EEG microstates – whole-brain patterns of dynamically evolving scalp potential topographies that reflect periods of synchronized neural activity – during both a go/no-go task and resting-state in 90, 4–8-year-old children. The mean amplitude of the ERN was quantified during the − 64 to 108 millisecond (ms) period of time relative to error commission, which was determined by data-driven microstate segmentation of error-related activity. We found that greater magnitude of the ERN associated with greater global explained variance (GEV; i.e., the percentage of total variance in the data explained by a given microstate) of an error-related microstate observed during the same − 64 to 108 ms period (i.e., error-related microstate 3), and to greater parent-report-measured anxiety risk. During resting-state, six data-driven microstates were identified. Both greater magnitude of the ERN and greater GEV values of error-related microstate 3 associated with greater GEV values of resting-state microstate 4, which showed a frontal-central scalp topography. Source localization results revealed overlap between the underlying neural generators of error-related microstate 3 and resting-state microstate 4 and canonical brain networks (e.g., ventral attention) known to support the higher-order cognitive processes involved in error processing. Taken together, our results clarify how individual differences in error-related and intrinsic brain activity are related and enhance our understanding of developing brain network function and organization supporting error processing during early childhood.

## 1. Introduction

The error-related negativity (ERN), a negative-going event-related potential (ERP) that peaks within 100 milliseconds (ms) of error commission at fronto-central scalp locations, is a psychophysiological marker of early performance monitoring associated with recognition and subsequent response to error (Gehring et al., 2012). In line with prior studies reporting marked changes in goal-directed behavior over the course of development, ERP research has similarly suggested that the ERN increases in strength from early childhood to adolescence and young adulthood (Boen et al., 2022; Tamnes et al., 2013). More specifically, a meta-analysis by Boen et al. (2022) recently reported a more negative ERN with increasing age in 3–28-year-olds with a small-to-medium effect size. As a result, maturational changes in the ERN have been suggested to potentially reflect the ongoing development of brain function and organization supporting error identification, processing, and subsequent goal-directed behavior (Boen et al., 2022; Tamnes et al., 2013). However, as observed in prior functional magnetic resonance imaging (fMRI) and electroencephalography (EEG) studies, error commission recruits a temporally evolving network of distributed and coordinated brain regions (Menon et al., 2001; Stevens et al., 2007; Völker et al., 2018) that may differ across development and are unlikely to be accurately captured by conventional ERP waveform analysis. That is, while the temporal (i.e., time window of interest) and spatial (i.e., electrodes of interest) assumptions generally used to inform *a priori* hypotheses concerning the ERN are likely to accurately capture local neural dynamics of error-related activity, they leave more global patterns potentially reflective of important developmental and/or individual-level differences largely underrepresented. As a result, how the ERN is associated with broader patterns of whole-brain activity supporting error processing remains unclear and needs further investigation using data-driven approaches that capitalize on the full temporal-spatial information available from EEG. Findings generated from these types of analyses are likely to be highly informative for understanding individual-level neurobiological differences in error processing and their association with developmental changes in behavior and cognition.

One multivariate, data-driven approach for characterizing the spatiotemporal dynamics of large-scale neural networks using EEG is *microstate analysis* (see Michel & Koenig, 2018 for a review). Microstates are patterns of scalp potential topographies, identified with clustering algorithms (e.g., *k*-means), that are stable in spatial location for very short periods of time (typically less than ~ 150 ms) and reflect rapidly evolving states of synchronized activity in the brain (Michel & Koenig, 2018). Microstate analysis can be performed on both task-evoked and resting-state data. As a result, the direct comparison of task-evoked and resting-state microstates using similarly defined units of measurement that characterize the temporal and spatial information of whole-brain activity is possible. Available temporal measures include global explained variance (GEV; percentage of total variance in the data explained by a given microstate), average duration in ms, coverage (i.e., percentage of recording time for which a given microstate is present), and occurrence (i.e., frequency per second; Michel & Koenig, 2018). While the nature of data collection is likely to shape the temporal properties of microstates identified during error processing or resting-state, as suggested by prior fMRI research demonstrating similar functional brain network structure and organization during task and rest (Cole et al., 2014; M. D. Fox & Raichle, 2007), a high degree of spatial overlap between individual microstates is expected given the significant likelihood of overlapping neural sources. Critically, if error-evoked activity and features of microstates identified at rest are associated as well, it may also be possible to predict the development of error processing and associated behavior and cognition from a much younger age when ERN data collection is not feasible (e.g., infants who are unable to perform tasks that elicit the ERN). As a result, understanding the associations between microstates generated using task versus resting-state data from the same individual children is highly likely to enhance our understanding of developing brain network function and organization supporting error processing.

Prior work in adults indicates that the time before and after error commission, time-locked to the behavioral response (e.g., button press), can be viewed as a sequence of a limited number of microstates with stable topographies that increase and decrease in strength or global field power (GFP; Britz & Michel, 2010; Pourtois, 2011; Vocat et al., 2008). Each microstate within the sequence reflects the activity of a different configuration of active populations of neurons whose electrical potentials propagate to the scalp through volume conduction (Michel & Koenig, 2018). As a result, the period of time immediately surrounding the peaks of conventional ERP components are almost always contained within a window of time represented by one of the identified microstates. When the group-level microstates from the grand-averaged, error-evoked data are fit back to the data of individual participants via spatial correlation (i.e., backfitting), individual differences in the temporal properties of each microstate become apparent across participants. As a result, a data-driven window of time for quantifying the given properties of an ERP of interest can be delineated, obviating the need to subjectively choose (either *a priori* or from grand-averaged waveforms) a window of time to investigate (e.g., measuring the ERN during the 0–100 ms period relative to error commission). Microstate analyses using resting-state EEG data have typically revealed 4–7 data-driven microstates that are remarkably highly spatially similar across participants and studies (Michel & Koenig, 2018). Importantly, simultaneous EEG-fMRI and EEG source imaging studies have demonstrated that the spatial patterns of EEG resting-state microstates resemble well-known fMRI resting-state networks (Bréchet et al., 2019; Britz et al., 2010; Custo et al., 2017). At the individual-level, prior work has also suggested that the temporal properties of each microstate (i.e., GEV, duration, coverage, and occurrence) show good internal consistency and test-retest reliability when as little as two minutes of resting-state data are used (Liu et al., 2020). Notably, the results of microstate analysis have also been shown to be unaffected by choice of the EEG reference (e.g., average or mastoids) (Michel & Koenig, 2018). This is particularly advantageous because previous research has shown differences in the ERN’s amplitude and quality depending on the reference used (Clayson et al., 2021). As a result, prior research suggests that systematically examining the associations between EEG microstates identified during task or at rest can be undertaken in a systematic and rigorous fashion at any age when this type of data can be collected.

In adults, data investigating how EEG microstates identified during rest are associated with those identified during error processing and to behavior are only beginning to emerge. More specifically, Kleinert et al. (2022) recently reported that fewer, but longer lasting microstates during rest (which according to the authors indicated more stable mental processing), regardless of microstate class, were associated with increased neural activity in the left insula and inferior frontal gyrus during a go/no-go task and with greater self-reported self-control. While to our knowledge no study has investigated microstates during error processing in young children, one recent study did assess awake resting-state EEG microstates exclusively during early childhood (Bagdasarov et al., 2022). More specifically, in a cross-sectional sample of 4–8-year-olds, we found sex-effects in the duration (i.e., males > females) of a microstate with an anterior-posterior orientation (i.e., microstate 3), and age by sex interaction effects for all of the temporal properties (i.e., generally, they decreased with increasing age for males but were constant for females) of a microstate with a centrally posterior orientation (i.e., microstate 4; Bagdasarov et al., 2022). Further, using source localization techniques, we found that microstates 3 and 4 were associated with brain networks previously identified in prior fMRI research to support higher-order cognitive functions (Bagdasarov et al., 2022). While these results suggest that similar microstates may be identified during error processing, and meaningfully related to neural markers of error-related activity, this has not been directly investigated in children. Therefore, given our recent resting-state EEG microstate findings and well documented changes in self-regulation during early childhood, an important next step is to directly investigate whether microstates identified during error processing and during rest are associated with each other and related to behavior in young children.

The current study aimed to investigate whether microstates during eyes-closed resting-state would associate with error-related neural activity (assessed using both microstate and ERP waveform analyses) during a go/no-go task in 90, 4–8-year-old children using high-density EEG. At the group-level, based on our prior work and that of others using a data-driven approach for microstate identification (Bagdasarov et al., 2022; Tomescu et al., 2018), we hypothesized that microstate analysis of resting-state EEG would produce 4–7 resting-state microstates. We also hypothesized, based on previous work on the ERN (Boen et al., 2022; Tamnes et al., 2013), that a microstate – which we will refer to as the *error-related microstate* hereafter – whose maximal activity would be at a fronto-central or central scalp location would define the peak of the ERN waveform surrounding error commission; thus, also defining the electrodes for which the ERN would be maximally negative. Further, we hypothesized that this microstate would have the highest spatial correlations with resting-state microstates that 1) have been suggested in previous work to represent brain networks involved in higher-order cognitive functions (Bagdasarov et al., 2022; Britz et al., 2010; Custo et al., 2017), and 2) have a central maximum along the longitudinal axis of the brain. At the individual-level, we hypothesized that an enhanced ERN during the period of time represented by the error-related microstate would be correlated with greater GEV values of the error-related microstate. We also hypothesized that both an enhanced ERN and greater GEV values of the error-related microstate would be correlated with greater GEV values of resting-state microstates that were highly spatially correlated with the error-related microstate. We chose GEV as our temporal parameter of interest because it represents the degree to which a particular microstate describes the dataset or time window of interest (i.e., it is easily interpretable), and can be compared between resting-state and task-evoked data (i.e., it measures the same property of neural activity in both types of data; i.e., goodness-of-fit or the sum of variances of the EEG data explained by a given microstate, normalized by GFP). Source localization of the error-related and resting-state microstates of interest was also carried out to identify overlapping patterns of brain activity. Given prior work reporting negative associations between ERN amplitude and measures of anxiety and anxiety risk in young children (Meyer, 2017), we hypothesized that an enhanced ERN would be correlated with greater parent-reported anxiety and behavioral inhibition. Lastly, following research suggesting that resting-state microstates defined by spatial topographies with central maxima along the longitudinal axis of the brain are associated with higher-order cognitive functions (Bagdasarov et al., 2022; Britz et al., 2010; Custo et al., 2017), we hypothesized that the GEV of these topographies would be correlated with greater parent-reported effortful control.

## 2. Methods

Participants were 90, 4–8-year-old children recruited from a database maintained by the Department of Psychology and Neuroscience at Duke University and community events. Recruitment details including inclusion and exclusion criteria are provided in the supplementary materials. All research was approved by Duke University’s Institutional Review Board and carried out in accordance with the Declaration of Helsinki. Caregivers provided informed consent and children provided verbal assent. Compensation was provided for study participation. Participant demographics are described in Table 1.

### 2.1. EEG Data Acquisition and Preprocessing

EEG was recorded at 1000 Hertz (Hz) and referenced to the vertex (channel Cz) using a 128-channel HydroCel Geodesic Sensor Net (Electrical Geodesics, Eugene, OR). Impedances were maintained below 50 kilohms throughout the EEG session. The resting-state paradigm consisted of eight one-minute blocks of alternating eyes-open and eyes-closed segments (i.e., four minutes of each). Following previous research, only the eyes-closed condition was analyzed for the current study (Bagdasarov et al., 2022; Tomescu et al., 2018). Event-related data was collected from a developmentally appropriate go/no-go paradigm (Chong & Meyer, 2019; Meyer et al., 2019; see supplementary materials for visualization). Children viewed images of aliens and astronauts as they appeared on a screen for 500 ms, followed by an intertrial interval, which was either 1000, 1500, or 2000 ms. They were instructed to “shoot” the aliens by pressing a button (“go” trials) and “save” the astronauts by withholding button-press (“no-go” trials). Pressing the button when an alien appeared was considered a correct “go” response while pressing the button when an astronaut appeared was considered an erroneous “no-go” response. After learning how to play the game during a practice round, children completed 120 “go” and 80 “no-go” trials. Both resting-state and go/no-go paradigms were presented with E-Prime software (Psychological Software Tools, Pittsburgh, PA).

Offline preprocessing was performed with EEGLAB (Delorme & Makeig, 2004) in MATLAB (MathWorks, Natick, MA). The preprocessing steps of continuous resting-state and task data overlapped but differed in some of their parameters. Details are provided in the supplementary materials and in custom scripts available on https://github.com/DEEDLabEEG. Briefly, 24 outer ring channels that often contain a large amount of artifact in developmental data were removed due to their location near the base of the skull or on the neck or face. Data were downsampled to 250 Hz, and bandpass filtered (1–40 Hz for resting; 0.1–40 Hz for task). After filtering, electrical line noise was still present in some participants’ data and attenuated using CleanLine (T. Mullen, 2012). Bad channels were removed and interpolated using spherical splines if they were 1) flat for more than 5 seconds, 2) contained more than 4 standard deviations of line noise relative to all other channels, or 3) correlated at less than .80 to surrounding channels. Following, data were re-referenced to the average. A copy of the original source data was created, cleaned using Artifact Subspace Reconstruction (ASR; T. R. Mullen et al., 2015; i.e., artifacted periods were removed using a burst criterion of 20 as recommended by Chang et al., 2020), and submitted to extended infomax independent component analysis (ICA; Lee et al., 1999) with principal component analysis (PCA) dimensionality reduction (i.e., 30 and 50 components, for resting and task data, respectively). The ICLabel plugin was used to flag components that had a probability of at least .70 of being related to eye or muscle artifacts (Pion-Tonachini et al., 2019). The resulting ICA fields and flags were subsequently copied over to the original source data and flagged ICA components were removed.

Resting-state data was segmented into nonoverlapping one-second epochs. Epochs were removed using the TBT plugin (Ben-Shachar, 2018) if at least 10 channels contained 1) amplitudes > 100 or < −100 μV, or 2) joint probabilities (i.e., probabilities of plausible activity) above 3 standard deviations for local or global thresholds. Correct “go” trials and erroneous “no-go” trials were segmented − 500 to 800 ms relative to the button-press, and baseline corrected during the − 500 to −300 ms period. The same epoch rejection criteria were applied, except amplitude-based rejection values were relaxed to > 150 or < −150 μV, and one additional criteria was added: epochs with at least 10 channels containing peak-to-peak amplitudes exceeding 100 μV within 200 ms windows sliding by 20 ms were also removed. For both types of data, if less than 10 channels met rejection criteria, the epoch was not removed, but the channels were interpolated for that epoch only. For task data, correct “go” trials and erroneous “no-go” trials were separately averaged for each participant after removing trials where the button-press occurred less than 100 ms after the stimulus or more than 2000 ms after the stimulus. A summary of the behavioral data (reaction time and accuracy means) is provided in the supplementary materials.

Data quality was rigorously assessed. Participants who did not pass EEG data quality checks were excluded from further analyses (see supplementary materials). Following exclusion of participants, the minimum amount of resting-state data across participants was 157 seconds. To reduce potential effects of varying data lengths on analyses, participants’ resting-state data were trimmed to their first 157 seconds, exceeding the previously published two-minute mark for the reliability of resting-state microstate analysis (Liu et al., 2020). For event-related data, following exclusion of participants, the minimum number of correct “go” and erroneous “no-go” trials was 21 and 6, respectively. Previous work has shown that the ERN can be reliability quantified using a minimum of 6 trials for each condition (Olvet & Hajcak, 2009).

### 2.2. Microstate Analysis

Microstate analysis of resting-state data was performed in Cartool (Brunet et al., 2011). First, at the individual-level, a spatial filter was applied to each participant’s data to remove topographic outliers and smooth topographies (Michel & Brunet, 2019). Topographies at global field power (GFP) peaks representing timepoints of the highest signal-to-noise ratio were extracted (Brunet et al., 2011). Fifty epochs of 833 random subsamples of each participant’s previously extracted topographies (covering 99.9% of each participant’s data) were submitted to a polarity-invariant modified *k*-means cluster analysis (Pascual-Marqui et al., 1995), which was set to repeat 50 times and identify 1–12 clusters of topographies for each epoch. The meta-criterion – an aggregate measure of seven independent criteria (Bréchet et al., 2019; Custo et al., 2017) – determined the optimal number of clusters for each epoch, resulting in *k* optimal clusters for each of 50 epochs. Next, at the group-level, the 50 sets of *k* optimal clusters from each participant were combined resulting in 4,500 sets (90 participants × 50 sets). 100 epochs each composed of 1000 randomly sampled sets (covering 98.8% of the sets) were submitted to a polarity-invariant modified *k*-means cluster analysis, which was set to repeat 100 times and identify 1–15 clusters of topographies for each epoch. The meta-criterion determined the optimal number of clusters for each epoch, resulting in *k* optimal clusters for each of 100 epochs. Lastly, these 100 sets were combined and submitted to a final polarity-invariant modified *k*-means cluster analysis, which was set to repeat 100 times and identify 1–15 clusters of topographies. The meta-criterion determined the optimal number of group-level resting-state microstates. The resampling approach is thought to improve the reliability of *k*-means clustering and has been used in recent work (Bagdasarov et al., 2022; Férat et al., 2022).

The group-level resting-state microstates were then backfitted to each participant’s original, spatially filtered data, including all data points (not just at GFP peaks). The data was normalized by the median of GFP to account for individual differences in scalp potential due to varying skull conductivity. Backfitting involved calculating the spatial correlation between each microstate at the group-level and each individual data point for each participant, such that the microstate with the highest correlation was assigned to that data point. The polarity of maps was ignored when calculating the correlation and the minimum correlation for data points assigned to a microstate was 50%. After backfitting, temporal smoothing (window half-size of 32 ms, Besag factor of 10; Pascual-Marqui et al., 1995) was applied, and improbably small segments were removed, such that segments smaller than 32 ms were divided in half with the first half added to the preceding segment and the second half added to the proceeding segment. The backfitting procedure produced values of each microstate’s GEV for each participant.

Microstate analysis of the grand-averaged (i.e., averaged across participants), spatially filtered, error-related activity was performed in one stage in Cartool. Error-related activity was calculated by subtracting correct “go” activity from erroneous “no-go” activity. Topographies at each timepoint (not just at GFP peaks), except for timepoints during the baseline period, were extracted and submitted to a modified *k*-means cluster analysis, which accounted for polarity, repeated 300 times, and identified 1–20 clusters of topographies. The meta-criterion determined the optimal number of clusters. Each timepoint of the grand-averaged data was then sequentially labeled with one of the topographies. The microstate surrounding the button-press – the *error-related microstate* – was identified by visual inspection of the segmented grand-averaged data. This microstate was backfitted to each participant’s original, spatially filtered, averaged, error-related activity using the same procedure as described for resting-state data; however, polarity was accounted for, the minimum spatial correlation was .25 instead of .50 (as done in Iannotti et al., 2022), and backfitting was only performed in the window of time represented by that particular microstate. The backfitting procedure produced values of this microstate’s GEV for each participant.

Reliability via internal consistency values were calculated for each microstate measure of interest.

### 2.3. Source Localization of Microstates

Six thousand solution points were distributed equally in a grey matter-constrained head model of a child MRI brain volume template. The EEG net template was co-registered to the MRI head model. The Local Spherical Model with Anatomical Constraints (LSMAC; Brunet et al., 2011) calculated an adaptive local spherical model at each electrode by estimating the thicknesses of the scalp, skull, cerebrospinal fluid, and brain under each electrode. These thicknesses were then used in a 4-shell spherical model with local radiuses, allowing the real geometry between solution points and electrodes to be accounted for. A distributed linear inverse solution, LORETA (Low Resolution Brain Electromagnetic Tomography; Pascual-Marqui et al., 1994), was calculated. The results were optimized with regularization, which accounted for background EEG noise and enforced smoothness of the results. The results were also standardized to correct for the variability of EEG power across time, a procedure automatically implemented in Cartool to eliminate activation biases (Michel & Brunet, 2019). The amplitude of dipoles produced for each solution point were saved as scalar, positive values at each solution point and averaged across timepoints for each microstate. For the resting-state microstates, only the norm of each dipole was saved. For the task microstate, the complete vector of each dipole was saved. Each microstate’s source map was thresholded to the solution points above the 95th percentile of values across participants (Bagdasarov et al., 2022; Bréchet et al., 2020, 2021). To facilitate interpretation of the potential functional significance of identified microstate sources and their overlap, source maps were converted to volumes and imported to the Analysis of Functional NeuroImages (AFNI; Cox, 1996) program. In AFNI, and for the resting-state microstates only, microstates were demeaned by subtracting the mean of all source maps from each source map to highlight microstate-specific sources (Bagdasarov et al., 2022; Custo et al., 2017). The center of mass coordinates for overlapping clusterized sources between resting-state and task microstates of interest were calculated. We also calculated how well microstates overlapped with seven canonical functional networks (Schaefer et al., 2018). Additional source localization details are provided in the supplementary materials.

### 2.4. Microstate-Guided ERN Waveform Analysis

The error-related microstate defined the window of time coinciding with the ERN (i.e., immediately prior to and following the button press) and channels (i.e., a pool of channels that were maximally negative). For each participant, the mean activity during the microstate-identified window of time and across microstate-identified channels was calculated separately for correct “go” and erroneous “no-go” trials. Reliability via internal consistency values were calculated using the ERP Reliability Analysis toolbox (Clayson & Miller, 2017). Following previous research, unstandardized residual values from a regression model in which participants’ correct “go” values were entered as the independent variable and erroneous “no-go” values were entered as the dependent variable were calculated (Meyer et al., 2017; see supplementary materials) and used in subsequent analyses. This procedure removed any variance in the erroneous “no-go” activity accounted for by correct “go” activity, which also tends to elicit a response similar to but smaller than the error-related response (Coles et al., 2001). We also calculated the ERN with other common scoring procedures and show high correlations between the values of all methods in the supplementary materials.

### 2.5. Parent-Report Questionnaires

#### Preschool Anxiety Scale (PAS)

The PAS is a 28-item parent-report measure of child anxiety during the preschool years (Spence et al., 2001). Each of the items are rated on a five-point scale from 0 representing “not at all” to 4 representing “very often true.” Factor analyses of the PAS have shown a five-factor model consistent with *Diagnostic and Statistical Manual of Mental Disorders, Fourth Edition* (DSM-IV; American Psychiatric Association, 1994) diagnostic categories for anxiety, including obsessive compulsive disorder, social anxiety, separation anxiety, physical injury fears, and generalized anxiety (Broeren & Muris, 2008; Spence et al., 2001). A higher order “anxiety” factor has been shown to explain the covariance between factors (Spence et al., 2001). PAS scales have shown moderate to high internal consistency values (alpha coefficients between .59 and .86). Further, PAS scales have shown to moderately correlate with internalizing symptoms and to correlate to a much lower extent with externalizing symptoms as measured by the Child Behavior Checklist (CBCL; Achenbach, 1999) (Broeren & Muris, 2008; Spence et al., 2001). A total score representing the sum of all items was calculated and used in analyses for the current study.

#### Behavioral Inhibition System and Behavioral Approach System (BIS/BAS) Scales, Parent-Report

The BIS/BAS scales are a 20-item measure of an individual’s withdraw and approach tendencies in response to environmental stimuli (Carver & White, 1994). The original BIS/BAS scales have been modified for use with parent-report for children (Blair, 2003; Blair et al., 2004; Muris et al., 2005). Each of the items are rated on a four-point scale from 0 representing “not true” to 3 representing “very true.” Factor analyses of the BIS/BAS scales in both adults and children have consistently demonstrated separate BIS and BAS factors (Bjørnebekk, 2009; Carver & White, 1994; Jorm et al., 1998; Muris et al., 2005; Ross et al., 2002). Only the seven items part of the BIS scale were summed and used in the current study. In adults, the BIS scale has demonstrated high internal consistency (alpha coefficient of .74; Carver & White, 1994). In children, the self-report version of the BIS scale has also demonstrated high internal consistency (alpha coefficients between .73 and .80 depending on the study; Bjørnebekk, 2009; Muris et al., 2005; Vervoort et al., 2010). Further, the BIS scale has shown to relate to but remain distinguishable from measures of anxiety (Carver & White, 1994).

#### Children’s Behavior Questionnaire, Short Form (CBQ-SF)

The CBQ-SF is a 94-item parent-report measure of temperament in early to middle childhood (Putnam & Rothbart, 2006). It was designed for research purposes to measure the same 15 domains of temperament as the CBQ with fewer items (Rothbart et al., 2001). Each of the items are rated on a seven-point scale from 1 representing “extremely untrue” to 7 representing “extremely true.” Factor analyses of the CBQ (Rothbart et al., 2001) and the CBQ-SF (de la Osa et al., 2014) have demonstrated a three-factor solution: extraversion/surgency, negative affectivity, and effortful control. Further, correlations between CBQ and CBQ-SF scores for extraversion/surgency, negative affectivity, and effortful control scales have been demonstrated to all be at or above .75 (Putnam & Rothbart, 2006). Only the effortful control scale was used in analyses for the current study. The effortful control scale was calculated by summing the items for attentional focusing, inhibitory control, and low-intensity pleasure subscales, which have been shown to have consistent factor affiliations with effortful control from infancy to early childhood (Putnam et al., 2008). Attentional focusing, inhibitory control, and low-intensity pleasure subscales from the CBQ-SF have shown moderate to high internal consistency values (alpha coefficients between .69 and .75; Putnam & Rothbart, 2006).

### 2.6. Data-Driven Selection of Resting-State Microstates of Interest

It was hypothesized that the spatial and temporal properties of the error-related microstate of interest (i.e., the microstate coinciding with the ERN) would relate to resting-state microstates that were similar in their spatial topographies. As such, a polarity-invariant spatial correlation between the error-related microstate of interest and each of the resting-state microstates was calculated to determine the resting-state microstates of interest for subsequent statistical analyses.

### 2.7. Statistical Approach

Hierarchical regression and linear regression analyses were performed in R (R Core Team, 2022). First, for each of eight models, multivariate outliers were identified by the Minimum Covariance Determinant and removed (Rousseeuw & Driessen, 1999). For hierarchical regression analyses (models 1–6), age and sex were entered in the first step. In the second step, the independent variable of interest was added (models 1, 2, 3 = residualized ERN amplitude; models 4 and 5 = error-related microstate 3 GEV; model 6 = resting-state microstate 4 GEV) to assess whether it significantly related to the dependent variable of interest after accounting for age and sex (model 1 = error-related microstate 3 GEV; models 2 and 4 = resting-state microstate 4 GEV; models 3 and 5 = resting-state microstate 6 GEV; model 6 = CBQ-SF effortful control scale). For linear regression analyses, residualized ERN amplitude was used to predict PAS (model 7) and BIS scores (model 8), separately. Regression assumptions were tested for all models, and corrective actions were taken if assumptions were violated (see supplementary materials). To minimize the Type I error rate, a Benjamini-Hochberg-correction for eight comparisons (i.e., the total number of models run) was applied to the *p* values of each omnibus *F* test of the final model, which tested the overall significance of the complete set of predictors (see supplementary materials). Effect sizes and post-hoc achieved power values were calculated for statistically significant models in G*Power (Faul et al., 2007).

## 3. Results

**Figure 1:**
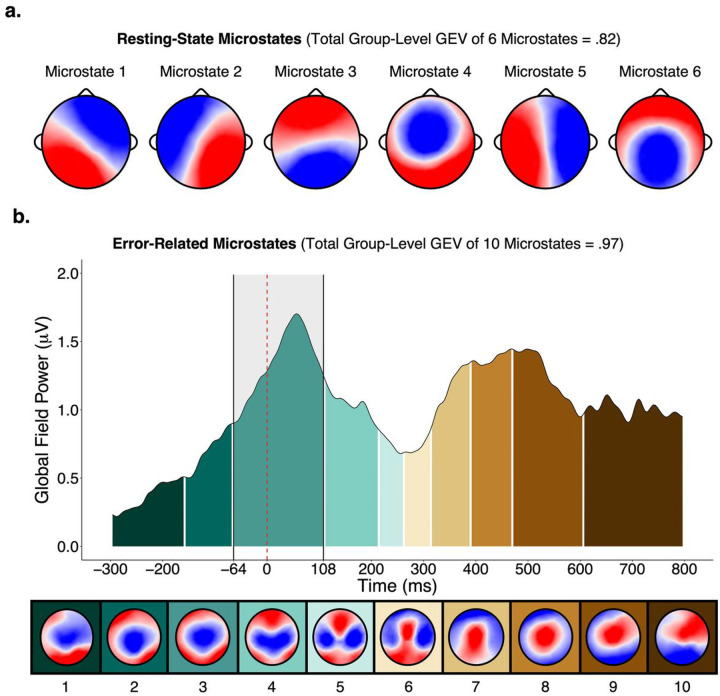
Resting-state and error-related microstates. *Note*. Resting-state microstates (a) were derived from a polarity-invariant clustering algorithm, while error-related microstates (b) were derived from a polarity-variant clustering algorithm. The dashed red line (b) represents the time of button-press. The highlighted shaded grey area (b) represents the time period of error related microstate 3. GEV = global explained variance.

**Figure 2:**
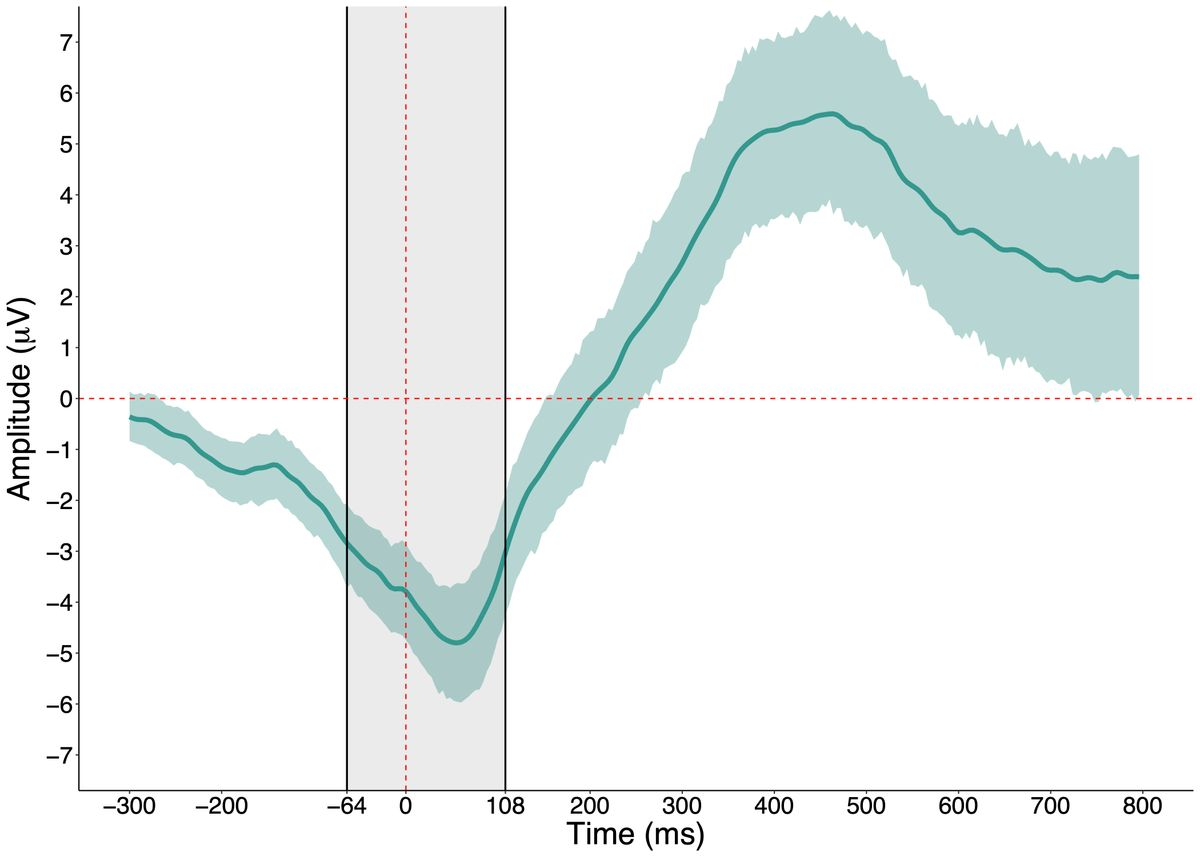
The ERN difference waveform at central scalp locations. *Note*. The dashed red line represents the time of button-press. The highlighted shaded grey area represents the time period of error related microstate 3. The ERN difference waveform was created by subtracting correct “go” from erroneous “no-go” activity. The highlighted shaded green area around the ERN difference waveform represents a bootstrapped 95% confidence interval.

The meta-criterion revealed six resting-state microstates (Fig. 1a) and 10 error-related microstates (Fig. 1b). The error-related microstate of interest was *error-related microstate 3*, which occurred from − 64 ms prior to and 108 ms following the button-press and coincided with the ERN. It was confirmed that the majority of participants had error-related microstate 3 during the − 64 to 108 ms period, and that error-related microstate 3 was present to a much smaller extent during other periods outside of this window (see supplementary materials). Visualization of the topography of error-related microstate 3 revealed maximally negative activity at central locations (i.e., Cz). As such, quantification of the ERN was performed for a pool of channels surrounding Cz (see supplementary materials for region-of-interest). Further, visualization of the grand-averaged difference ERN waveform across participants confirmed that its most negative peak was contained within the − 64 to 108 ms period (Fig. 2). Reliability of all neural measures are provided in the supplementary materials. Spatial correlations revealed that resting-state microstates 4 and 6 had the highest topographic similarity with error-related microstate 3 (correlations of .80 and .51, respectively) while the other microstates were highly dissimilar (correlations below .20; see supplementary materials). Descriptive statistics of the temporal parameters of each microstate and questionnaire data are presented in the supplementary materials. Given ongoing debate about how to select the optimal number of resting-state microstates – whether selection should be based on previous research, objective data-driven methods, or some combination thereof – spatial correlations between the topographies of four- and six-state solutions and correlations between their temporal parameters are provided in the supplementary materials as well.

### 3.1. Regression Analyses

Results of regression analyses are summarized below. Additional details are provided in the supplementary materials, including results with outliers included in models (neither the significance of models nor direction of effects change with outliers included).

#### 3.1.1. Relationship between residualized ERN amplitude and error-related microstate 3 GEV (model 1; n = 84).

**Figure 3:**
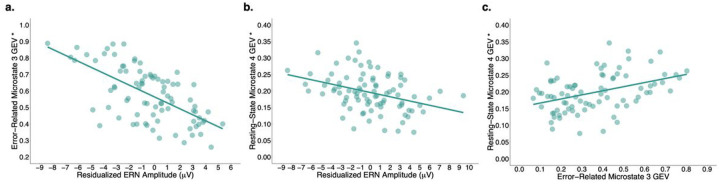
Partial residuals plots showing relationships between (a) residualized ERN amplitude and error-related microstate 3 GEV, (b) residualized ERN amplitude resting-state microstate 4 GEV, and (c) error-related microstate 3 GEV and resting-state microstate 4 GEV. *Note*.Asterisk (*) indicates that the variable was square root transformed (see supplementary materials).

The addition of residualized ERN amplitude to age and sex was associated with a statistically significant increment to the proportion of variation in error-related microstate 3 GEV explained, Δ Adj. *R*^2^ = .39, *F*(2, 80) = 51.75, *p* < .001, partial adj. *R*^2^ = .39, *f*^2^ = .64, power > .99. The final model containing all three predictors explained a significant proportion of the observed variation in error-related microstate 3 GEV, Adj. *R*^2^ = .38, *F*(3, 80) = 18.02, corrected *p* < .001, *f*^2^ = .61, power > .99. Results indicated that a one standard deviation increase in residualized ERN amplitude associated with a .63 standard deviation decrease in error-related microstate 3 GEV, all else held constant, *t*(80) = −7.18, *p* < .001, 95% CI [−0.05, −0.03] (Fig. 3a).

#### 3.1.2. Relationship between residualized ERN amplitude and resting-state microstate 4 GEV (model 2; n = 88).

The addition of residualized ERN amplitude to age and sex was associated with a statistically significant increment to the proportion of variation in resting-state microstate 4 GEV explained, Δ Adj. *R*^2^ = .08, *F*(2, 84) = 12.20, *p* < .001, partial adj. *R*^2^ = .12, *f*^2^ = .14, power = .93. The final model containing all three predictors explained a significant proportion of the observed variation in resting-state microstate 4 GEV, Adj. *R*^2^ = .38, *F*(3, 84) = 18.60, corrected *p* < .001, *f*^2^ = .61, power > .99. Results indicated that a one standard deviation increase in residualized ERN amplitude associated with a .30 standard deviation decrease in resting-state microstate 4 GEV, all else held constant, *t*(84) = −3.50, *p* < .001, 95% CI [−0.01, −0.003] (Fig. 3b).

#### 3.1.3. Relationship between error-related microstate 3 GEV and resting-state microstate 4 GEV (model 3; n = 82).

The addition of error-related microstate 3 GEV to age and sex was associated with a statistically significant increment to the proportion of variation in resting-state microstate 4 GEV explained, Δ Adj. *R*^2^ = .11, *F*(2, 78) = 15.00, *p* < .001, partial adj. *R*^2^ = .15, *f*^2^ = .18, power = .96. The final model containing all three predictors explained a significant proportion of the observed variation in resting-state microstate 4 GEV, Adj. *R*^2^ = .38, *F*(3, 84) = 18.60, corrected *p* < .001, *f*^2^ = .61, power > .99. Results indicated that a one standard deviation increase in error-related microstate 3 GEV associated with a .34 standard deviation increase in resting-state microstate 4 GEV, all else held constant, *t*(78) = 3.88, *p* < .001, 95% CI [0.06, 0.19] (Fig. 3c).

#### 3.1.4. Relationship between residualized ERN amplitude and resting-state microstate 6 GEV (model 4; n = 86).

The addition of residualized ERN amplitude to age and sex was not associated with a statistically significant increment to the proportion of variation in resting-state microstate 6 GEV explained, Δ Adj. *R*^2^ = − .01, *F*(2, 83) = 0.36, *p* = .55. The final model containing all three predictors explained a significant proportion of the observed variation in resting-state microstate 6 GEV, Adj. *R*^2^ = .12, *F*(3, 83) = 4.78, corrected *p* = .01, *f*^2^ = .14, power = .81. However, results indicated that residualized ERN amplitude was not associated with resting-state microstate 6 GEV, all else held constant, *t*(83) = 0.60, *p* = .55, 95% CI [−0.004, 0.01].

#### 3.1.5. Relationship between error-related microstate 3 GEV and resting-state microstate 6 GEV (model 5; n = 82).

The addition of error-related microstate 3 GEV to age and sex was not associated with a statistically significant increment to the proportion of variation in resting-state microstate 6 GEV explained, Δ Adj. *R*^2^ = .01, *F*(2, 78) = 0.01, *p* = .94. The final model containing all three predictors explained a significant proportion of the observed variation in resting-state microstate 6 GEV, Adj. *R*^2^ = .14, *F*(3, 78) = 5.24, corrected *p* = .004, *f*^2^ = .16, power = .86. However, results indicated that error-related microstate 3 GEV was not associated with resting-state microstate 6 GEV, all else held constant, *t*(78) = −0.08, *p* = .94, 95% CI [−0.09, 0.08].

#### 3.1.6. Relationship between resting-state microstate 4 GEV and CBQ-SF effortful control scores (model 6; n = 87).

The addition of resting-state microstate 4 GEV to sex was not associated with a statistically significant increment to the proportion of variation in CBQ-SF effortful control scores explained, Δ Adj. *R*^2^ = − .01, *F*(1, 84) = 0.15, *p* = .70. The final model containing all two predictors did not explain a significant proportion of the observed variation in CBQ-SF effortful control scores, Adj. *R*^2^ = .03, *F*(2, 84) = 2.17, corrected *p* = .14. Results indicated that resting-state microstate 4 GEV was not associated with CBQ-SF effortful control, all else held constant, *t*(84) = 0.38, *p* = .70, 95% CI [−4.73, 7.00].

#### 3.1.7. Relationship between residualized ERN amplitude and PAS scores (model 7; n = 86).

Residualized ERN amplitude did not explain a significant proportion of the observed variation in PAS scores, Adj. *R*^2^ = − .01, *F*(1, 84) = 0.08, corrected *p* = .99.

#### 3.1.8. Relationship between residualized ERN amplitude and BIS scores (model 8; n = 88).

Residualized ERN amplitude explained a significant proportion of the observed variation in BIS scores, Adj. *R*^2^ = .10, *F*(1, 86) = 10.60, corrected *p* = .003, *f*^2^ = .11, power = .87. Results indicated that a one standard deviation increase in residualized ERN amplitude associated with a .33 standard deviation decrease in BIS scores, *t*(86) = −3.25, *p* = .002, 95% CI [−1.10, −0.27] (plot in supplementary materials).

### 3.2. Sources

**Figure 4:**
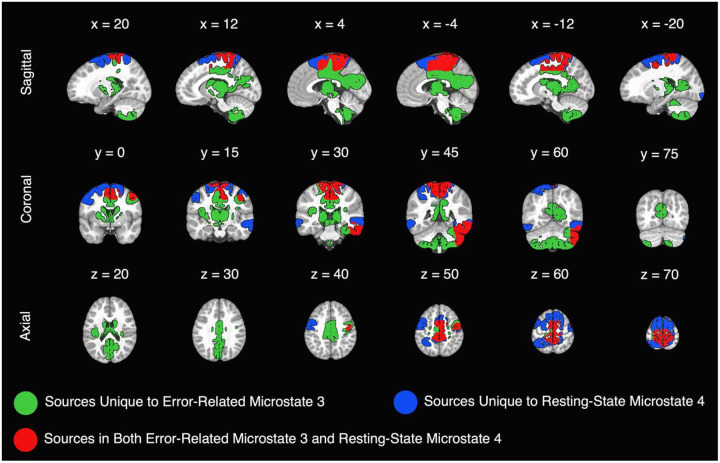
Neural sources of resting-state microstate 4 and error-related microstate 3. *Note*. Sagittal slices (x plane) are presented as left (positive coordinates) to right (negative coordinates) parts of the brain. Coronal slices (y plane) are presented as anterior (negative coordinates) to posterior (positive coordinates) parts of the brain. Axial slices (z plane) are presented as inferior to superior parts of the brain.

Source localization results are presented in Fig. 4. Overlap between identified sources for error-related microstate 3 and resting-state microstate 4 are presented in both Fig. 4 and Table 2. In addition to the sources listed in Table 2 from center of mass analyses, the following sources were also observed in both error-related microstate 3 and resting-state microstate 4: Left precentral gyrus, bilateral paracentral lobules, bilateral superior frontal gyri, bilateral cingulate gyri, bilateral precuneus, left fusiform gyrus, and right inferior temporal gyrus. Table 3 presents how well the sources of each microstate overlapped with seven canonical functional networks. The somatomotor, dorsal attention, and ventral attention networks overlapped most with resting-state microstate 4 sources. The somatomotor, ventral attention, and visual networks overlapped most with error-related microstate 3 sources. When combined, somatomotor, dorsal attention, and ventral attention networks best represented sources in resting-state microstate 4 and error-related microstate 3. The somatomotor, visual, and ventral attention networks best represented sources that overlapped between resting-state microstate 4 and error-related microstate 3.

## 4. Discussion

The current study investigated the association between EEG microstate spatial topographies and related properties during resting-state and error-related activity in a large sample of 4–8-year-old children. *A priori* defined quantitative criteria indicated that a sequence of 10 unique microstates characterized EEG data collected during a go/no-go task and that six microstates were representative of the unique spatial topographies present in resting-state EEG data. Of the 10 error-related microstates identified, error-related microstate 3 captured the peak of the ERN waveform and was most topographically similar to resting-state microstates 4 and 6, being most similar to resting-state microstate 4. Using regression analyses, an enhanced ERN was found to be associated with greater GEV values of error-related microstate 3 and resting-state microstate 4. Further, greater GEV values of error-related microstate 3 were associated with greater GEV values of resting-state microstate 4. Conversely, neither ERN nor GEV values of error-related microstate 3 were associated with GEV values of resting-state microstate 6. Source localization of resting-state microstate 4 and error-related microstate 3 identified overlapping patterns of brain activity in canonical functional networks shown in prior fMRI research to support higher-order cognitive processes (e.g., ventral attention network). Further, an enhanced ERN was associated with greater parent-reported child behavioral inhibition but not anxiety symptoms. There was no association between GEV values of resting-state microstate 4 and effortful control as measured by parent-report. Overall, results show promise for understanding how individual differences in error-related and intrinsic brain activity during early childhood may be related to each other and to behavior and cognition. They also provide, for the first time, information about the functional significance of resting-state microstates during early childhood.

Supporting error-related microstate 3 as a global measure of distributed and coordinated brain regions involved in error commission processes, we found that greater error-related microstate 3 GEV values were associated with an enhanced ERN (i.e., a stronger psychophysiological response to error commission). As in prior research, ERN amplitude in the current study measured neural activity localized to a central region of the scalp where error-related activity was maximal. While useful for understanding local neural dynamics, measuring error-related activity in this way is unlikely to fully capture the spatially and temporally evolving network of brain regions involved in error processing. As discussed previously, a microstate analytic approach overcomes this limitation and allows for the identification and measurement of unique spatiotemporal topographies that capture global neural dynamics across the entire scalp during error-related processing. Given that an association between data extracted from these two approaches would be anticipated (i.e., global encompasses local), it is not surprising that the current study found individual differences in the ERN to be associated with those of a specific topographical representation of error-related activity across the entire scalp (i.e., error microstate 3). However, while expected, by directly demonstrating this association the current study supports the use of task-related microstates to further define the functional properties of those identified in the absence of an explicit task (e.g., resting-state). And, as a result, the current findings are a critical step forward for informing future EEG microstate studies of error-related brain function and organization in developmental (e.g., infants) or psychiatric (e.g., autism spectrum disorder) groups unable to successfully perform and/or comply with explicit tasks.

Both an enhanced ERN and greater GEV values of error-related microstate 3 associated with greater GEV values of resting-state microstate 4 in our sample of young children. That is, individual differences in both the local and global neural dynamics of error processing were reflected in individual differences in resting-state data, and vice versa. In an overlapping, but not identical sample of participants, we previously found that a microstate with a central maximum along the longitudinal axis of the brain represented brain networks involved in higher-order cognitive functions (Bagdasarov et al., 2022). This microstate showed that its temporal parameters decreased with age, but only in males, potentially reflecting previously published behavioral and observational data showing maturational differences in the development of neurocognitive systems, including those responsible for executive functions, between sexes (Berlin & Bohlin, 2002; Brocki & Bohlin, 2004; Carlson & Moses, 2001; Zelazo et al., 2008; Zelazo & Carlson, 2012). Taken together with the observed relationship between GEV values of resting-state microstate 4 and error-related data, the current study suggests that resting-state microstates can be used to measure developmental changes in neurobiological correlates of specific higher-order psychological constructs (e.g., those indexing error processing or performance monitoring) as early as the preschool period. However, future research investigating the associations between EEG microstates identified during rest and systematic measurements of relevant behavior (e.g., orienting and/or sustaining attention) will be necessary to establish the potential functional specificity of any microstate more fully whether identified at rest or during task.

Source localization of resting-state microstate 4 and error-related microstate 3 revealed that overlap in neural generators was greatest for the canonical somatomotor network. The somatomotor network, which includes regions of the brain involved in sensory processing, motor planning, and motor execution, was most represented in the overlap of sources from resting-state and error-related data as shown in Tables 2 and 3. In line with this finding, previous work using fMRI in adults demonstrated that error-related activity elicited activation in a network of somatomotor areas (Hester et al., 2004), and that the amplitude of the ERN related to the strength of functional connectivity between the dorsal anterior cingulate cortex (dACC) and motor regions of the brain (Gilbertson et al., 2021). Interestingly, when examining the sources of error-related microstate 3 and resting-state microstate 4 individually, an area within the dACC was only present for error-related microstate 3. As a result, findings from the current study suggest that somatomotor activity is involved in neural response to error in young children, potentially through connectivity with other task-induced patterns of neural activity (e.g., dACC). Importantly, they also suggest that the overlapping somatomotor regions shared between error-related and resting-state microstates may act as part of a larger network of regions involved in cognitive control that can also be identified using resting-state data(Gordon et al., 2023). However, future research will be required to directly investigate the presence and potential role of somatomotor cortex across multiple tasks requiring coordinated network activity. The next largest percentage of overlap in sources for resting-state microstate 4 and error-related microstate 3 was found in the ventral attention network (Table 3). Functionally, the ventral attention network plays an important role in the detection and reorientation of attention towards salient stimuli in the environment (Corbetta & Shulman, 2002). When an error is made, the ventral attention network is involved in the detection of mismatch between expected and actual outcomes. Following error, the ventral attention network is involved in the reorientation of attention toward task-relevant information. Therefore, the overlap in ventral attention network sources of resting-state microstate 4 and error-related microstate 3 further supports the observed positive relationship between their temporal properties and suggests that microstates observed during rest can index higher-order cognitive functions involving systems of attention. Interestingly, while the percentage of voxels found within the ventral attention network was similar for each microstate when examined in isolation, resting-state microstate 4 also had sources located within parts of the dorsal attention network. This difference in the representation of dorsal and ventral attention networks in the sources of error-related microstate 3 and resting-state microstate 4 may reflect the complementary roles of each network. The dorsal attention network is responsible for top-down, goal-directed control of attention while the ventral attention network is responsible for bottom-up, stimuli-driven control of attention (Corbetta & Shulman, 2002). As such, the dorsal attention network may be better represented in microstates from resting-state data as children work to maintain focus of their attention inward and not respond to distraction (i.e., attention is directed by the experimenter to internal thoughts while participants sit still with their eyes closed). In contrast, intrinsic activity of the ventral attention network during resting-state may be directly involved in the activity of the ventral attention network during performance monitoring tasks as children detect and orient to salient stimuli (i.e., aliens and astronauts in our go/no-go paradigm). Future work that manipulates children’s internal and external states of attention should clarify the representation and roles of dorsal and ventral attention networks in EEG microstates.

Given a large and growing body of work showing support for the relationship between ERN amplitude and measures of anxiety and anxiety risk in children, we conducted regression analyses to assess whether the ERN’s amplitude related to parent-report measures of anxiety symptoms and behavioral inhibition. Our results revealed that the relationship between the ERN and individual differences in anxiety and anxiety risk is complex. While an enhanced ERN associated with greater levels of behavioral inhibition, a known risk factor for the future development of anxiety in children and adults (Rosenbaum et al., 1993; Sandstrom et al., 2020), the ERN showed no relationship with anxiety symptoms. This discrepant finding, which was contrary to our hypotheses, suggests that our measures of behavioral inhibition and anxiety likely quantified related but distinct psychological phenomena, and that the ERN in our sample may be a more specific marker of behavioral inhibition than of anxiety. In fact, the BIS scale assessed children’s sensitivity to negative outcomes and their tendency to inhibit their behavior in response to potential threat or punishment (Carver & White, 1994). On the other hand, the PAS measured the number and severity of children’s anxiety symptoms (Spence et al., 2001). Therefore, the BIS scale measured a temperamental trait while the PAS measured a mental state that is likely influenced by factors beyond temperament. In addition, the ERN during early childhood may reflect a neurobiological vulnerability to anxiety that is not yet expressed as specific symptoms but is captured in measures of anxiety risk, such as the BIS scale. It may also be possible that sample characteristics – a normative, non-clinically anxious sample of participants – were responsible for null findings between the ERN and specific anxiety symptoms. Previous work examining the relationship between the ERN and normative variations in anxiety found that the strength and direction of their association changed across childhood. For example, in non-clinically anxious younger children, the ERN was blunted, while in non-clinically anxious older children and adolescents the ERN was enhanced (Meyer, 2017). Given the relatively narrow range of participants’ ages in the current study (i.e., 4–8 years), it was not possible to assess a similar moderating role of age, but it is likely an important factor to consider in future work.

Effortful control – the ability to inhibit a dominant response in order to perform a subdominant response, to detect errors, and to engage in planning – relies on attentional resources to move, focus, and sustain attention as needed (Rueda, 2012). Although it is well established that attentional processes are involved in the execution of effortful control, contrary to our hypothesis, we did not find a relationship between GEV values of resting-state microstate 4 and parent-reported effortful control. However, while a cross-sectional relationship did not exist, GEV values of resting-state microstate 4 may relate to the development of effortful control over time. Additional research is necessary to examine this longitudinal possibility.

Finally, we found that neither the amplitude of the ERN nor GEV values of error-related microstate 3 associated with GEV values of resting-state microstate 6. One hypothesized explanation for this pattern of results may be the high spatial correlation (.80) between error-related microstate 3 and resting-state microstate 4, but moderate spatial correlation (.51) between error-related microstate 3 and resting-state microstate 6. Similar to the pattern of source localization results observed with resting-state microstate 4, overlap in the neural generators of resting-state microstate 6 and error-related microstate 3 was greatest for the somatomotor network (see supplementary materials). However, overlap in somatomotor activity was comparatively smaller for resting-state microstate 6, suggesting that somatomotor sources known to be involved in error processing were present to a smaller extent. Further, results revealed that resting-state microstate 6 and error-related microstate 3 did not overlap in other canonical networks, demonstrating the potentially unique functional role of resting-state microstate 4 in error processing.

### 4.1. Strengths, Limitations, and Future Directions

The current study is the first to investigate the functional significance of EEG microstates during early childhood, and the first to directly compare microstates from task-evoked and resting-state data; an important step toward understanding how related, but distinct pieces of information about the brain from resting-state and task data can inform our developmental understanding of network function and organization. Despite the relatively lower spatial resolution of EEG when compared to fMRI, EEG source localization aided in the interpretation of microstate results. Viewing microstates within a framework of well-studied fMRI-derived functional networks has the potential to clarify how dynamically evolving spatiotemporal properties of the brain may relate to individual differences in behavior. One limitation of this approach, however, is that EEG source analyses are not currently able to distinguish patterns of activation versus deactivation. This limits the interpretation of our result to a rudimentary quantification of the presence or absence of sources. The present sources of any given microstate likely represent patterns of both activation and deactivation, which we cannot discern as well. Further while the structure of resting-state networks is similar between children and adults (Bie et al., 2012; Muetzel et al., 2016; Supekar et al., 2009), future work should use reliable and age-appropriate network parcellations as they become available to increase the precision of source localization results and their interpretation. Despite these limitations, the rich temporal information derived from EEG is unparalleled and provides unique and reliable information that can further our developmental understanding of how functional brain networks rapidly change and develop in children. Future use of individual MRI scans and EEG channel coordinate location as well as multimodal EEG-fMRI studies may additionally enrich our understanding of how related or distinct EEG microstates are to fMRI resting-state networks in children.

In addition to characterizing global patterns of electrical activity, we used microstate analysis to provide an objective, data-driven window of time for which the mean amplitude of the ERN was measured. This is a unique advantage over other approaches that rely on previous research or subjective inspection of waveform peaks and scalp topographies to determine measurement windows. While beyond the scope of the currents study, it may be important for future work to examine the relationship between ERPs measured during traditional windows of time compared to those derived from the results of microstate segmentation. In fact, different measurement windows may quantify distinct neurobiological phenomena (e.g., wider windows may capture neural activity from greater regions of the brain representing the evolving networks of sources during error processing), and selection of one over the other may impact brain-behavior relationships.

Not unique to the current study, microstate analysis relies on the group-level backfitting of microstates to participant-level data. While the derivation of group-level microstates considers the data of all participants, it may not be the best approach for assessing individual differences between the temporal parameters of microstates and individual differences in behavior. Future work should try to understand whether group-level segmentation and backfitting impacts brain-behavior relationships. For example, our null findings between the GEV values of resting-state microstate 4 and effortful control may have been the result of the loss of important microstate topographies during group-level microstate segmentation. If group-level segmentation and backfitting impacts brain-behavior relationships, then new methods using the microstates approach should be developed to better assess individual differences in microstate topographies.

While cross-sectional, the results of the current study show promise for investigating potential longitudinal relationships between resting-state and error-related activity. From the current study, we know that both the amplitude of the ERN and GEV values of error-related microstate 3 relate to resting-state microstate 4. Therefore, a longitudinal relationship may exist between resting-state microstate 4 measured during infancy and error-related microstate 3 measured during the preschool and school-age years. If so, we may be able to predict the development of error processing from a much younger age when resting-state, but not error-related EEG data can be reliably collected. In addition, since behavioral inhibition shows relative continuity across the lifespan from infancy through adulthood (N. A. Fox et al., 2005), it may be possible to predict neurobiological risk for anxiety with resting-state data before measurement of the ERN is possible (e.g., infancy), and many years before symptoms of anxiety begin to emerge. As a result, early interventions can be more effectively implemented at younger ages to mitigate risk.

### 4.2. Conclusion

In a young sample of 90, 4–8-year-old children, we identified associations between the ERN and whole-brain patterns of error-related and resting-state brain activity using EEG microstate analyses. As a result, the current study provides novel insights into how error-related and resting-state EEG microstate are associated with each other and how this type of approach can be used to further clarify the functional significance of resting-state microstates. Future longitudinal research is now needed to build on these findings and to enhance our neurodevelopmental understanding of error processing as early as infancy.

## Figures and Tables

**Table 1:** Participant demographics (*n* = 90).

	Mean (SD)	Range
Age (years)	6.49 (1.10)	4.51–8.96
Income-to-Needs Ratio *	3.06 (1.03)	0.69–4.69
	*n*	Percent (%)
Biological Sex		
Females	52	57.78
Males	38	42.22
Race		
White	65	72.22
Multiracial	14	15.56
Black or African American	7	7.78
Asian	2	2.22
Other	2	2.22
Native Hawaiian or Other Pacific Islander	0	0
Ethnicity		
Not Hispanic or Latino	78	86.67
Hispanic or Latino	12	13.33
Maternal Education		
Some Grade School	0	0
Completed Grade School	0	0
Some High School	0	0
High School Diploma	1	1.11
Some College or 2-Year Degree	20	22.22
4-Year College Degree	27	30
Some School Beyond College	1	1.11
Professional or Graduate Degree	41	45.56

*Note*. The Income-to-Needs Ratio (ITN) was calculated by dividing total family income by a poverty threshold determined by the United States Census Bureau, which considered the year assessed and household family size. One participant did not have ITN information available. Participant demographics for the reduced *n* = 84 sample (see Results section) are provided in the supplementary materials.

**Table 2:** Center of mass coordinates for overlapping error-related microstate 3 and resting-state microstate 4 clusterized sources.

Cluster	Voxels	x	y	z	Talairach-Tournoux Atlas Location	Schaefer Network *
1	44038	−1.2	27.9	62.6	Right Medial Frontal Gyrus	Somatomotor
2	31705	−48.5	45.5	−26.7	Right Fusiform Gyrus	Visual
3	4688	−46.8	7.4	48.3	Right Precentral Gyrus	Somatomotor
4	583	−18.4	0.2	57.0	Right Medial Frontal Gyrus	Dorsal Attention
5	324	−50.4	30.7	−6.7	Right Middle Temporal Gyrus	Default
6	55	−46.5	19.0	46.4	Right Postcentral Gyrus	Dorsal Attention
7	32	−17.7	9.5	61.1	Right Middle Frontal Gyrus	Dorsal Attention
8	30	−22.0	36.2	55.9	Right Postcentral Gyrus	Somatomotor
9	16	−21.0	12.9	53.1	Right Middle Frontal Gyrus	Dorsal Attention
10	6	−35.7	30.5	−11.2	Right Parahippocampal Gyrus	Visual
11	4	16.5	14.0	58.5	Left Medial Frontal Gyrus	Dorsal Attention
12	1	26.0	37.0	49.0	Left Postcentral Gyrus	Somatomotor

*Note*. Clusters organized from largest to smallest in volume. Clusterization performed in AFNI with the following parameters: Level 3 clustering method (i.e., faces or edges or corners) with minimum cluster size of 1 voxel.

* Schaefer-Yeo AFNI 2021 parcellation (7 networks, 100-area).

**Table 3:** Percentage of voxels from source localization of microstates that are part of seven canonical functional networks.

Functional Network	Resting-State Microstate 4 Sources	Error-Related Microstate 3 Sources	Resting-State Microstate 4 Sources + Error-Related Microstate 3 Sources	Overlapping Sources Between Resting-State Microstate 4 & Error-Related Microstate 3
Visual	7.04%	14.64%	17.38%	4.30%
Somatomotor	25.29%	26.87%	32.91%	19.24%
Dorsal Attention	21.72%	5.57%	23.33%	3.97%
Ventral Attention	16.63%	15.05%	23.41%	8.26%
Limbic	4.67%	1.85%	5.07%	1.46%
Control	2.30%	8.82%	9.75%	1.38%
Default	10.82%	8.43%	16.43%	2.82%

*Note*. Percentages represent the mathematical intersection of localized microstate sources and functional network sources from the Schaefer-Yeo AFNI 2021 parcellation (7 networks, 100-area). Percentages for each column do not add up to 100% because sources may be located outside of canonical networks.

## Data Availability

The data that support the findings of this study are available on request from the corresponding author. The data are not publicly available due to privacy or ethical restrictions.

## References

[1] Achenbach T. M. (1999). The Child Behavior Checklist and related instruments. In The use of psychological testing for treatment planning and outcomes assessment, 2nd ed (pp. 429–466). Lawrence Erlbaum Associates Publishers.

[2] American Psychiatric Association. (1994). Diagnostic and Statistical Manual of Mental Disorders: DSM-IV. American Psychiatric Association.

[3] Bagdasarov A., Roberts K., Bréchet L., Brunet D., Michel C. M., & Gaffrey M. S. (2022). Spatiotemporal dynamics of EEG microstates in four- to eight-year-old children: Age- and sex-related effects. Developmental Cognitive Neuroscience, 57, 101134. 10.1016/j.dcn.2022.101134 35863172 PMC9301511

[4] Ben-Shachar M. S. (2018). TBT: Reject and interpolate channels on a epoch by epoch basis (2.6.1). 10.5281/zenodo.1241518

[5] Berlin L., & Bohlin G. (2002). Response Inhibition, Hyperactivity, and Conduct Problems Among Preschool Children. Journal of Clinical Child & Adolescent Psychology, 31(2), 242–251. 10.1207/S15374424JCCP3102_09 12056107

[6] de Bie H. M. A., Boersma M., Adriaanse S., Veltman D. J., Wink A. M., Roosendaal S. D., Barkhof F., Stam C. J., Oostrom K. J., de Waal H. A. D., & Sanz-Arigita E. J. (2012). Resting-state networks in awake five- to eight-year old children. Human Brain Mapping, 33(5), 1189–1201. 10.1002/hbm.21280 21520347 PMC6870031

[7] Bjørnebekk G. (2009). Psychometric Properties of the Scores on the Behavioral Inhibition and Activation Scales in a Sample of Norwegian Children. Educational and Psychological Measurement, 69(4), 636–654. 10.1177/0013164408323239

[8] Blair C. (2003). Behavioral inhibition and behavioral activation in young children: Relations with self-regulation and adaptation to preschool in children attending Head Start. Developmental Psychobiology, 42(3), 301–311. 10.1002/dev.10103 12621656

[9] Blair C., Peters R., & Granger D. (2004). Physiological and neuropsychological correlates of approach/withdrawal tendencies in preschool: Further examination of the behavioral inhibition system/behavioral activation system scales for young children. Developmental Psychobiology, 45(3), 113–124. 10.1002/dev.20022 15505800

[10] Boen R., Quintana D. S., Ladouceur C. D., & Tamnes C. K. (2022). Age-related differences in the error-related negativity and error positivity in children and adolescents are moderated by sample and methodological characteristics: A meta-analysis. Psychophysiology, 59(6), e14003. 10.1111/psyp.14003 35128651 PMC9285728

[11] Bréchet L., Brunet D., Birot G., Gruetter R., Michel C. M., & Jorge J. (2019). Capturing the spatiotemporal dynamics of self-generated, task-initiated thoughts with EEG and fMRI. NeuroImage, 194, 82–92. 10.1016/j.neuroimage.2019.03.029 30902640

[12] Bréchet L., Brunet D., Perogamvros L., Tononi G., & Michel C. M. (2020). EEG microstates of dreams. Scientific Reports, 10(1), Article 1. 10.1038/s41598-020-74075-z PMC7553905 33051536

[13] Bréchet L., Ziegler D. A., Simon A. J., Brunet D., Gazzaley A., & Michel C. M. (2021). Reconfiguration of Electroencephalography Microstate Networks after Breath-Focused, Digital Meditation Training. Brain Connectivity, 11(2), 146–155. 10.1089/brain.2020.0848 33403921 PMC7984939

[14] Britz J., & Michel C. M. (2010). Errors can be related to pre-stimulus differences in ERP topography and their concomitant sources. NeuroImage, 49(3), 2774–2782. 10.1016/j.neuroimage.2009.10.033 19850140

[15] Britz J., Van De Ville D., & Michel C. M. (2010). BOLD correlates of EEG topography reveal rapid resting-state network dynamics. NeuroImage, 52(4), 1162–1170. 10.1016/j.neuroimage.2010.02.052 20188188

[16] Brocki K. C., & Bohlin G. (2004). Executive Functions in Children Aged 6 to 13: A Dimensional and Developmental Study. Developmental Neuropsychology, 26(2), 571–593. 10.1207/s15326942dn2602_3 15456685

[17] Broeren S., & Muris P. (2008). Psychometric evaluation of two new parent-rating scales for measuring anxiety symptoms in young Dutch children. Journal of Anxiety Disorders, 22(6), 949–958. 10.1016/j.janxdis.2007.09.008 17977693

[18] Brunet D., Murray M. M., & Michel C. M. (2011). Spatiotemporal Analysis of Multichannel EEG: CARTOOL. Computational Intelligence and Neuroscience, 2011, e813870. 10.1155/2011/813870 PMC3022183 21253358

[19] Carlson S. M., & Moses L. J. (2001). Individual Differences in Inhibitory Control and Children’s Theory of Mind. Child Development, 72(4), 1032–1053. 10.1111/1467-8624.00333 11480933

[20] Carver C. S., & White T. L. (1994). Behavioral inhibition, behavioral activation, and affective responses to impending reward and punishment: The BIS/BAS Scales. Journal of Personality and Social Psychology, 67, 319–333. 10.1037/0022-3514.67.2.319

[21] Chang C.-Y., Hsu S.-H., Pion-Tonachini L., & Jung T.-P. (2020). Evaluation of Artifact Subspace Reconstruction for Automatic Artifact Components Removal in Multi-Channel EEG Recordings. IEEE Transactions on Biomedical Engineering, 67(4), 1114–1121. 10.1109/TBME.2019.2930186 31329105

[22] Chong L. J., & Meyer A. (2019). Understanding the Link between Anxiety and a Neural Marker of Anxiety (The Error-Related Negativity) in 5 to 7 Year-Old Children. Developmental Neuropsychology, 44(1), 71–87. 10.1080/87565641.2018.1528264 30407088

[23] Clayson P. E., Baldwin S. A., Rocha H. A., & Larson M. J. (2021). The data-processing multiverse of event-related potentials (ERPs): A roadmap for the optimization and standardization of ERP processing and reduction pipelines. NeuroImage, 245, 118712. 10.1016/j.neuroimage.2021.118712 34800661

[24] Clayson P. E., & Miller G. A. (2017). ERP Reliability Analysis (ERA) Toolbox: An open-source toolbox for analyzing the reliability of event-related brain potentials. International Journal of Psychophysiology, 111, 68–79. 10.1016/j.ijpsycho.2016.10.012 27769878

[25] Cole M. W., Bassett D. S., Power J. D., Braver T. S., & Petersen S. E. (2014). Intrinsic and task-evoked network architectures of the human brain. Neuron, 83(1), 238–251. 10.1016/j.neuron.2014.05.014 24991964 PMC4082806

[26] Coles M. G. H., Scheffers M. K., & Holroyd C. B. (2001). Why is there an ERN/Ne on correct trials? Response representations, stimulus-related components, and the theory of error-processing. Biological Psychology, 56(3), 173–189. 10.1016/S0301-0511(01)00076-X 11399349

[27] Corbetta M., & Shulman G. L. (2002). Control of goal-directed and stimulus-driven attention in the brain. Nature Reviews Neuroscience, 3(3), 201–215. 10.1038/nrn755 11994752

[28] Cox R. W. (1996). AFNI: Software for Analysis and Visualization of Functional Magnetic Resonance Neuroimages. Computers and Biomedical Research, 29(3), 162–173. 10.1006/cbmr.1996.0014 8812068

[29] Custo A., Van De Ville D., Wells W. M., Tomescu M. I., Brunet D., & Michel C. M. (2017). Electroencephalographic Resting-State Networks: Source Localization of Microstates. Brain Connectivity, 7(10), 671–682. 10.1089/brain.2016.0476 28938855 PMC5736178

[30] de la Osa N., Granero R., Penelo E., Domènech J. M., & Ezpeleta L. (2014). The Short and Very Short Forms of the Children’s Behavior Questionnaire in a Community Sample of Preschoolers. Assessment, 21(4), 463–476. 10.1177/1073191113508809 24235176

[31] Delorme A., & Makeig S. (2004). EEGLAB: An open source toolbox for analysis of single-trial EEG dynamics including independent component analysis. Journal of Neuroscience Methods, 134(1), 9–21. 10.1016/j.jneumeth.2003.10.009 15102499

[32] Faul F., Erdfelder E., Lang A.-G., & Buchner A. (2007). G*Power 3: A flexible statistical power analysis program for the social, behavioral, and biomedical sciences. Behavior Research Methods, 39(2), 175–191. 10.3758/BF03193146 17695343

[33] Férat V., Seeber M., Michel C. M., & Ros T. (2022). Beyond broadband: Towards a spectral decomposition of electroencephalography microstates. Human Brain Mapping, 1–15. 10.1002/hbm.25834 PMC9188972 35324021

[34] Fox M. D., & Raichle M. E. (2007). Spontaneous fluctuations in brain activity observed with functional magnetic resonance imaging. Nature Reviews. Neuroscience, 8(9), 700–711. 10.1038/nrn2201 17704812

[35] Fox N. A., Henderson H. A., Marshall P. J., Nichols K. E., & Ghera M. M. (2005). Behavioral Inhibition: Linking Biology and Behavior within a Developmental Framework. Annual Review of Psychology, 56(1), 235–262. 10.1146/annurev.psych.55.090902.141532 15709935

[36] Gehring W. J., Liu Y., Orr J. M., & Carp J. (2012). The error-related negativity (ERN/Ne). In The Oxford handbook of event-related potential components (pp. 231–291). Oxford University Press.

[37] Gilbertson H., Fang L., Andrzejewski J. A., & Carlson J. M. (2021). Dorsal anterior cingulate cortex intrinsic functional connectivity linked to electrocortical measures of error monitoring. Psychophysiology, 58(5), e13794. 10.1111/psyp.13794 33624288 PMC8049966

[38] Gordon E. M., Chauvin R. J., Van A. N., Rajesh A., Nielsen A., Newbold D. J., Lynch C. J., Seider N. A., Krimmel S. R., Scheidter K. M., Monk J., Miller R. L., Metoki A., Montez D. F., Zheng A., Elbau I., Madison T., Nishino T., Myers M. J., … Dosenbach N. U. F. (2023). A somato-cognitive action network alternates with effector regions in motor cortex. Nature, 1–9. 10.1038/s41586-023-05964-2 37076628 PMC10172144

[39] Hester R., Fassbender C., & Garavan H. (2004). Individual Differences in Error Processing: A Review and Reanalysis of Three Event-related fMRI Studies Using the GO/NOGO Task. Cerebral Cortex, 14(9), 986–994. 10.1093/cercor/bhh059 15115734

[40] Iannotti G. R., Orepic P., Brunet D., Koenig T., Alcoba-Banqueri S., Garin D. F. A., Schaller K., Blanke O., & Michel C. M. (2022). EEG Spatiotemporal Patterns Underlying Self-other Voice Discrimination. Cerebral Cortex, 32(9), 1978–1992. 10.1093/cercor/bhab329 34649280 PMC9070353

[41] Jorm A. F., Christensen H., Henderson A. S., Jacomb P. A., Korten A. E., & Rodgers B. (1998). Using the BIS/BAS scales to measure behavioural inhibition and behavioural activation: Factor structure, validity and norms in a large community sample. Personality and Individual Differences, 26(1), 49–58. 10.1016/S0191-8869(98)00143-3

[42] Kleinert T., Nash K., Leota J., Koenig T., Heinrichs M., & Schiller B. (2022). A Self-Controlled Mind is Reflected by Stable Mental Processing. PsyArXiv. 10.31234/osf.io/fzg9y 36279561

[43] Lee T.-W., Girolami M., & Sejnowski T. J. (1999). Independent Component Analysis Using an Extended Infomax Algorithm for Mixed Subgaussian and Supergaussian Sources. Neural Computation, 11(2), 417–441. 10.1162/089976699300016719 9950738

[44] Liu J., Xu J., Zou G., He Y., Zou Q., & Gao J.-H. (2020). Reliability and Individual Specificity of EEG Microstate Characteristics. Brain Topography, 33(4), 438–449. 10.1007/s10548-020-00777-2 32468297

[45] Menon V., Adleman N. E., White C. d., Glover G. h., & Reiss A. l. (2001). Error-related brain activation during a Go/NoGo response inhibition task. Human Brain Mapping, 12(3), 131–143. 10.1002/1097-0193(200103)12:3<131::AID-HBM1010>3.0.CO;2-C 11170305 PMC6872006

[46] Meyer A. (2017). A biomarker of anxiety in children and adolescents: A review focusing on the error-related negativity (ERN) and anxiety across development. Developmental Cognitive Neuroscience, 27, 58–68. 10.1016/j.dcn.2017.08.001 28818707 PMC6987910

[47] Meyer A., Carlton C., Chong L. J., & Wissemann K. (2019). The Presence of a Controlling Parent Is Related to an Increase in the Error-Related Negativity in 5–7 Year-Old Children. Journal of Abnormal Child Psychology, 47(6), 935–945. 10.1007/s10802-018-0503-x 30610550

[48] Meyer A., Lerner M. D., De Los Reyes A., Laird R. D., & Hajcak G. (2017). Considering ERP difference scores as individual difference measures: Issues with subtraction and alternative approaches. Psychophysiology, 54(1), 114–122. 10.1111/psyp.12664 28000251

[49] Michel C. M., & Brunet D. (2019). EEG Source Imaging: A Practical Review of the Analysis Steps. Frontiers in Neurology, 10, 325. 10.3389/fneur.2019.00325 31019487 PMC6458265

[50] Michel C. M., & Koenig T. (2018). EEG microstates as a tool for studying the temporal dynamics of whole-brain neuronal networks: A review. NeuroImage, 180, 577–593. 10.1016/j.neuroimage.2017.11.062 29196270

[51] Muetzel R. L., Blanken L. M. E., Thijssen S., van der Lugt A., Jaddoe V. W. V., Verhulst F. C., Tiemeier H., & White T. (2016). Resting-state networks in 6-to-10 year old children. Human Brain Mapping, 37(12), 4286–4300. 10.1002/hbm.23309 27417416 PMC6867466

[52] Mullen T. (2012). NITRC: CleanLine: Tool/Resource Info. https://www.nitrc.org/projects/cleanline

[53] Mullen T. R., Kothe C. A. E., Chi Y. M., Ojeda A., Kerth T., Makeig S., Jung T.-P., & Cauwenberghs G. (2015). Real-time neuroimaging and cognitive monitoring using wearable dry EEG. IEEE Transactions on Biomedical Engineering, 62(11), 2553–2567. 10.1109/TBME.2015.2481482 26415149 PMC4710679

[54] Muris P., Meesters C., de Kanter E., & Timmerman P. E. (2005). Behavioural inhibition and behavioural activation system scales for children: Relationships with Eysenck’s personality traits and psychopathological symptoms. Personality and Individual Differences, 38(4), 831–841. 10.1016/j.paid.2004.06.007

[55] Olvet D. M., & Hajcak G. (2009). The stability of error-related brain activity with increasing trials. Psychophysiology, 46(5), 957–961. 10.1111/j.1469-8986.2009.00848.x 19558398

[56] Pascual-Marqui R. D., Michel C. M., & Lehmann D. (1994). Low resolution electromagnetic tomography: A new method for localizing electrical activity in the brain. International Journal of Psychophysiology, 18(1), 49–65. 10.1016/0167-8760(84)90014-X 7876038

[57] Pascual-Marqui R. D., Michel C. M., & Lehmann D. (1995). Segmentation of brain electrical activity into microstates: Model estimation and validation. IEEE Transactions on Biomedical Engineering, 42(7), 658–665. 10.1109/10.391164 7622149

[58] Pion-Tonachini L., Kreutz-Delgado K., & Makeig S. (2019). ICLabel: An automated electroencephalographic independent component classifier, dataset, and website. NeuroImage, 198, 181–197. 10.1016/j.neuroimage.2019.05.026 31103785 PMC6592775

[59] Pourtois G. (2011). Early Error Detection Predicted by Reduced Pre-response Control Process: An ERP Topographic Mapping Study. Brain Topography, 23(4), 403–422. 10.1007/s10548-010-0159-5 20717842

[60] Putnam S. P., & Rothbart M. K. (2006). Development of Short and Very Short Forms of the Children’s Behavior Questionnaire. Journal of Personality Assessment, 87(1), 102–112. 10.1207/s15327752jpa8701_09 16856791

[61] Putnam S. P., Rothbart M. K., & Gartstein M. A. (2008). Homotypic and heterotypic continuity offline-grained temperament during infancy, toddlerhood, and early childhood. Infant and Child Development, 17(4), 387–405. 10.1002/icd.582

[62] R Core Team. (2022). R: a Language and Environment for Statistical Computing. R Foundation for Statistical Computing. https://www.R-project.org/

[63] Rosenbaum J. F., Biederman J., Bolduc-Murphy E. A., Faraone S. V., Chaloff J., Hirshfeld D. R., & Kagan J. (1993). Behavioral Inhibition in Childhood: A Risk Factor for Anxiety Disorders. Harvard Review of Psychiatry, 1(1), 2–16. 10.3109/10673229309017052 9384823

[64] Ross S. R., Millis S. R., Bonebright T. L., & Bailley S. E. (2002). Confirmatory factor analysis of the Behavioral Inhibition and Activation Scales. Personality and Individual Differences, 33(6), 861–865. 10.1016/S0191-8869(01)00196-9

[65] Rothbart M. K., Ahadi S. A., Hershey K. L., & Fisher P. (2001). Investigations of Temperament at Three to Seven Years: The Children’s Behavior Questionnaire. Child Development, 72(5), 1394–1408. 10.1111/1467-8624.00355 11699677

[66] Rousseeuw P. J., & Driessen K. V. (1999). A Fast Algorithm for the Minimum Covariance Determinant Estimator. Technometrics, 41(3), 212–223. 10.1080/00401706.1999.10485670

[67] Rueda M. R. (2012). Effortful control. In Handbook of temperament (pp. 145–167). The Guilford Press.

[68] Sandstrom A., Uher R., & Pavlova B. (2020). Prospective Association between Childhood Behavioral Inhibition and Anxiety: A Meta-Analysis. Research on Child and Adolescent Psychopathology, 48(1), 57–66. 10.1007/s10802-019-00588-5 31642030

[69] Schaefer A., Kong R., Gordon E. M., Laumann T. O., Zuo X.-N., Holmes A. J., Eickhoff S. B., & Yeo B. T. T. (2018). Local-Global Parcellation of the Human Cerebral Cortex from Intrinsic Functional Connectivity MRI. Cerebral Cortex (New York, N.Y.: 1991), 28(9), 3095–3114. 10.1093/cercor/bhx179 28981612 PMC6095216

[70] Spence S. H., Rapee R., McDonald C., & Ingram M. (2001). The structure of anxiety symptoms among preschoolers. Behaviour Research and Therapy, 39(11), 1293–1316. 10.1016/S0005-7967(00)00098-X 11686265

[71] Stevens M. C., Kiehl K. A., Pearlson G. D., & Calhoun V. D. (2007). Brain network dynamics during error commission. Human Brain Mapping, 30(1), 24–37. 10.1002/hbm.20478 PMC2669663 17979124

[72] Supekar K., Musen M., & Menon V. (2009). Development of Large-Scale Functional Brain Networks in Children. PLOS Biology, 7(7), e1000157. 10.1371/journal.pbio.1000157 19621066 PMC2705656

[73] Tamnes C. K., Walhovd K. B., Torstveit M., Sells V. T., & Fjell A. M. (2013). Performance monitoring in children and adolescents: A review of developmental changes in the error-related negativity and brain maturation. Developmental Cognitive Neuroscience, 6, 1–13. 10.1016/j.dcn.2013.05.001 23777674 PMC6987843

[74] Tomescu M. I., Rihs T. A., Rochas V., Hardmeier M., Britz J., Allali G., Fuhr P., Eliez S., & Michel C. M. (2018). From swing to cane: Sex differences of EEG resting-state temporal patterns during maturation and aging. Developmental Cognitive Neuroscience, 31, 58–66. 10.1016/j.dcn.2018.04.011 29742488 PMC6969216

[75] Vervoort L., Wolters L. H., Hogendoorn S. M., de Haan E., Boer F., & Prins P. J. M. (2010). Sensitivity of Gray’s Behavioral Inhibition System in clinically anxious and non-anxious children and adolescents. Personality and Individual Differences, 48(5), 629–633. 10.1016/j.paid.2009.12.021

[76] Vocat R., Pourtois G., & Vuilleumier P. (2008). Unavoidable errors: A spatio-temporal analysis of time-course and neural sources of evoked potentials associated with error processing in a speeded task. Neuropsychologia, 46(10), 2545–2555. 10.1016/j.neuropsychologia.2008.04.006 18533202

[77] Völker M., Fiederer L. D. J., Berberich S., Hammer J., Behncke J., Kršek P., Tomášek M., Marusič P., Reinacher P. C., Coenen V. A., Helias M., Schulze-Bonhage A., Burgard W., & Ball T. (2018). The dynamics of error processing in the human brain as reflected by high-gamma activity in noninvasive and intracranial EEG. NeuroImage, 173, 564–579. 10.1016/j.neuroimage.2018.01.059 29471099

[78] Zelazo P. D., & Carlson S. M. (2012). Hot and Cool Executive Function in Childhood and Adolescence: Development and Plasticity. Child Development Perspectives, 6(4), 354–360. 10.1111/j.1750-8606.2012.00246.x

[79] Zelazo P. D., Carlson S. M., & Kesek A. (2008). The development of executive function in childhood. In Handbook of developmental cognitive neuroscience, 2nd ed (pp. 553–574). MIT Press.

